# Identification of subclusters and prognostic genes based on GLS-associated molecular signature in ulcerative colitis

**DOI:** 10.1038/s41598-024-63891-2

**Published:** 2024-06-07

**Authors:** Yang Xie, Jun Li, Qing Tao, Yonghui Wu, Zide Liu, Youxiang Chen, Chunyan Zeng

**Affiliations:** Department of Gastroenterology, Digestive Disease Hospital, The First Affiliated Hospital, Jiangxi Medical College, Nanchang University, Nanchang, China

**Keywords:** Ulcerative colitis, Machine-learning algorithms, Diagnostic markers, Immune cell infiltration, Machine learning, Immunology, Gastroenterology, Medical research

## Abstract

Ulcerative colitis (UC) is a chronic and recurrent inflammatory disease that affects the colon and rectum. The response to treatment varies among individuals with UC. Therefore, the aim of this study was to identify and explore potential biomarkers for different subtypes of UC and examine their association with immune cell infiltration. We obtained UC RNA sequencing data from the GEO database, which included the training set GSE92415 and the validation set GSE87473 and GSE72514. UC patients were classified based on GLS and its associated genes using consensus clustering analysis. We identified differentially expressed genes (DEGs) in different UC subtypes through a differential expression analysis of the training cohort. Machine learning algorithms, including Weighted Gene Co-Expression Network Analysis (WGCNA), Least Absolute Shrinkage and Selection Operator (LASSO), and Support Vector Machine Recursive Feature Elimination (SVM-RFE), were utilized to identify marker genes for UC. The CIBERSORT algorithm was used to determine the abundance of various immune cells in UC and their correlation with UC signature genes. Finally, we validated the expression of GLS through in vivo and ex vivo experiments. The expression of GLS was found to be elevated in patients with UC compared to normal patients. GLS and its related genes were able to classify UC patients into two subtypes, C1 and C2. The C1 subtype, as compared to the C2 subtype, showed a higher Mayo score and poorer treatment response. A total of 18 DEGs were identified in both subtypes, including 7 up-regulated and 11 down-regulated genes. Four UC signature genes (CWH43, HEPACAM2, IL24, and PCK1) were identified and their diagnostic value was validated in a separate cohort (AUC > 0.85). Furthermore, we found that UC signature biomarkers were linked to the immune cell infiltration. CWH43, HEPACAM2, IL24, and PCK1 may serve as potential biomarkers for diagnosing different subtypes of UC, which could contribute to the development of targeted molecular therapy and immunotherapy for UC.

## Introduction

Ulcerative colitis (UC) is a chronic inflammatory bowel disease characterized by inflammation and ulceration of the colon and rectum^1^. It affects millions of people worldwide, with a higher prevalence in developed countries^2^. Current treatment options for UC mainly include medications such as amino salicylates, corticosteroids, immunomodulators, and biologics^2^. These therapeutic agents aim to control inflammation, induce and maintain remission, and prevent relapse. However, despite the availability of these treatments, a considerable proportion of UC patients still experience inadequate disease control, adverse effects, or loss of response over time^3^.

Glutamine is an important component of amino acid metabolism and is involved in the synthesis and degradation of amino acids in the body, maintains the body's nitrogen balance, participates in energy production, regulates the immune response, and fights oxidative stress, among other important roles^4–6^. Several previous studies have reported that glutamine is essential for tumor cell function, including the synthesis of metabolites that maintain mitochondrial metabolism, the generation of antioxidants to scavenge reactive oxygen species, the synthesis of non-essential amino acids (NEAAs), purines, pyrimidines, and fatty acids required for cellular replication, and the activation of cell signaling^4^. Targeting glutamine metabolism can inhibit the growth of a variety of tumor cells^7,8^, such as prostate cancer^9^, pancreatic cancer^10^, ovarian cancer^11^, breast cancer^12,13^, and lung cancer^14^. In addition, glutamine is involved in various metabolic pathways in cancer stem cells, such as the redox pathway, ATP generation, biosynthesis of non-essential amino acids and nucleotides, as well as ammonia production and epigenetic modification^15^. However, glutamine has been less frequently reported in UC. Glutamine was found to protect the intestinal mucosa by promoting mucosal cell growth and repair^16,17^. Further studies by Jeong et al. found that glutamine ameliorated DSS-induced colonic inflammation in mice by inducing the MAPK signaling pathway^18^. Similarly, a study by Giris et al. found that the intestinal mucosal structure was preserved in the glutamine-treated group, and that glutamine attenuated the damage to the colon caused by TNBS-induced colitis^19^. Interestingly, Bernd Sido et al. found that glutamine supplementation prevented inflammatory tissue damage in experimental ileitis. However, immune enhancement of colonic inflammatory cells by glutamine increased oxidative tissue damage^20^. These studies suggest that whether glutamine has a therapeutic effect in colitis remains controversial.

Glutaminase (GLS) is the first key enzyme that catalyzes the conversion of glutamine to glutamate, which plays a crucial role in cellular metabolism and energy production. GLS expression has been found to be elevated and pro-cancer in many tumors, such as breast cancer^21^, colorectal cancer^22^ and prostate cancer^23^. However, there are no studies related to the role and specific mechanisms of glutaminase in ulcerative colitis.

The aim of this study was to understand the role of GLS in UC, to determine the significance of GLS-related genes for typing in UC, and to identify signature genes in different subtypes of UC. In this study, we used machine learning algorithms to investigate and validate the characterized genes in patients with different subtypes of UC. In addition, we quantified immune cell infiltration in different subtypes of UC using the CIBERSORT algorithm, revealing the correlation between UC signature gene expression and immune cell infiltration. Gene set enrichment analysis (GSEA) was used to identify potentially relevant signaling pathways in different subtypes of UC. Finally, we verified the expression of GLS in acute DSS mouse model tissues and LPS-induced enteritis NCM460 cell model by western blotting (WB).

## Materials and methods

### GEO datasets selection and GLS-related genes

We conducted a systematic search for ulcerative colitis in the GEO database, focusing on colon samples from patients with ulcerative colitis and datasets containing relevant clinical information. After excluding animal studies, RNA-seq datasets, and whole blood samples, we identified several studies. The GSE92415 dataset included expression profiles from tissue samples of 162 UC patients and 21 healthy controls, while the GSE87473 dataset included expression profiles from tissue samples of 106 UC patients and 21 normal controls. Dataset GSE72514 comprises transcriptomic data obtained from colonic tissue samples collected from 97 UC patients. GSE92415 was designated as the discovery cohort, GSE87473 and GSE72514 was designated as the validation cohort. We identified GLS-related genes from the STRING database (https://string-db.org) based on protein interactions and signaling pathways.

### Consensus clustering analysis

Consensus cluster analysis is an unsupervised machine learning algorithm used to identify groups of samples that remain clustered together^24^. The R package "ConensusClusterPlus" was utilized to classify the UC samples in GSE92415 into different subtypes. The clustering process was repeated 10 times to ensure the confidence of the classification. The optimal number of clusters (k) was selected by examining the consistency matrix for each k-value and using the cumulative density function (CDF) of the consistency index. Following clustering, the different subtypes were visualized using principal component analysis and cluster analysis.

### Differently expressed genes (DEGs) determination

DEGs between cluster1 and cluster2 samples were identified using the Limma package in Rstudio. Criteria for DEGs included ∣Log2FC∣ > 1, p < 0.05, and a false discovery rate (FDR) < 0.05. FDR quantifies the proportion of false discoveries among a set of hypothesis tests deemed significant.

### Weighted gene co-expression network analysis (WGCNA) and functional enrichment analysis

WGCNA is a systems biology method used to identify co-expression modules, which are groups of genes jointly regulated under diverse conditions^25^. In our study, we utilized the WGCNA package in R to construct a co-expression network for GSE92415. Gene expression profiles were used to calculate correlations between gene pairs, resulting in a neighbor-joining matrix. Hierarchical clustering was then used to group highly correlated genes into co-expression modules. Modules containing more than 50 genes were identified using a hierarchical clustering tree method. These modules were subsequently correlated with external phenotypic data to identify relevant ones. We also identified core genes within each module that may play pivotal roles in specific biological processes or diseases. The gene ontology (GO) database^26^, developed by the Gene Ontology Consortium, serves as a standardized semantic vocabulary for characterizing and annotating the functions of genes and proteins across various species, with ongoing updates reflecting advances in research. GO annotations are categorized into three main domains: molecular functions (MF), biological processes (BP), and cellular components (CC). Kyoto Encyclopedia of Genes and Genomes (KEGG)^27–29^ is an extensive database that amalgamates genomic, chemical, and systemic functional data. It specializes in housing gene pathway information across diverse species. In this study, enrichment analyses, including GO and KEGG, were conducted on the modular genes using the clusterProfiler package in R. Significantly enriched pathways were determined by p < 0.05.

### GSEA functional enrichment analysis

The Gene Set Enrichment Analysis (GSEA) method relies on the concept of utilizing a pre-defined set of genes, typically derived from functional annotations or previous experimental findings, to rank genes based on their expression disparities across two sample types^30^. Subsequently, it evaluates whether this pre-defined gene set exhibits enrichment at either end of the ranked list. In this study, we utilized the clusterProfiler^31^ package to conduct GSEA analysis on the gene expression profile of dataset GSE92415. Specifically, we employed "c2.cp.kegg.v7.4.symbols.gmt" and "c5.go.bp.v7.4.symbols.gmt" as reference gene sets^32^, with significance defined at a threshold of p < 0.05.

### Characteristic biomarker identification by machine learning and validation

The Support Vector Machine Recursive Feature Elimination (SVM-RFE) algorithm, developed by Guyon et al. in 2002, is a machine learning approach used to identify tumor-specific genes. This method iteratively removes the least significant feature from a sorted list until the remaining features meet specified criteria^33^. SVM is a supervised machine learning method commonly employed for classification and regression tasks. To prevent overfitting, the Recursive Feature Elimination (RFE) algorithm is applied to select the optimal genes from the feature pool. Therefore, SVM-RFE technique is employed to identify the subset of genes with the highest discriminatory capability. Introduced by Tibshirani in 1996, the LASSO algorithm establishes a linear model linking input features and phenotypes by minimizing the sum of least squares loss and the L1 penalty term. LASSO, as a dimensionality reduction method, excels in handling high-dimensional data compared to regression analysis. The SVM-RFE and LASSO algorithms were independently implemented on the GEO training cohort using the R packages "e1071" and "glmnet". The common characteristic genes identified by both SVM-RFE and LASSO were extracted and presented in a Venn diagram using the "venn" package. Subsequently, the expression levels of these characteristic genes underwent validation in an independent validation cohort. To assess the classification performance of key genes between cluster1 and cluater2 samples, ROC curves and AUCs were computed using the "pROC" package in R. Statistical analyses were conducted using R version 4.3.0 and Prism software (GraphPad Prism 8.0, USA).

### Evaluation of immune cell infiltration

The CIBERSORT computational approach (http://cibersort.stanford.edu/) relies on a deconvolution algorithm for gene expression, enabling the assessment of genetic variation within a sample relative to the entire genome. The CIBERSORT algorithm, developed by Newman et al. in 2015, is a computational tool for deconvoluting expression matrices of human immune cell subtypes^34^. Based on linear support vector regression principles, it allows for the estimation of immune cell abundances within a given expression matrix^34^. In our study, we used the expression matrix derived from UC samples in the GSE92415 dataset as input for the CIBERSORT algorithm, enabling the calculation of immune cell abundances. The "vioplot" R package was employed to depict the distributions of the 22 immune cells in Cluster1 and Cluster2. Heatmaps illustrating the quantitative correlations among different immune cells were generated using the "corrplot" package. Additionally, the "ggplot2" R package facilitated the exploration of the relationship between the expression of diagnostic markers and the proportions of immune cells. We conducted statistical comparisons of immune cell infiltration levels across UC subtypes using Wilcoxon rank sum tests and visually represented the results through violin plots. To elucidate the relationship between characteristic gene expression and immune cell infiltration levels, we applied Spearman correlation analysis, and the results are depicted in lollipop plots.

### Mouse model of ulcerative colitis

A mouse model of chronic colitis was constructed using DSS, following previously described methods^35^. Normal control mice were given distilled water, while the experimental group received DSS. After 7 days of DSS treatment, all mice were anesthetized with 1% sodium pentobarbital (3 ml/kg) and euthanized by cervical dislocation. Subsequently, mice were dissected and pertinent tissues, such as the colon, were harvested for subsequent biological, immunological, or molecular experiments. All animal experiments were carried out according to the Guidelines for the Care and Use of Laboratory Animals.

### Cell culture

The normal human intestinal epithelial mucosa cell line (NCM460) was obtained from the Chinese Academy of Sciences (Shanghai, China). NCM460 cells were cultured in Gibco's RPMI 1640 medium (Grand Island, NY, USA) supplemented with 10% fetal bovine serum (FBS) from Gibco, and 1% penicillin–streptomycin from Solarbio (Beijing, China). The cell cultures were maintained in a controlled environment, and incubated at 37 °C with 5% CO_2_.

### Construction of the cellular inflammation model

To establish a model of inflammation in human intestinal epithelial cells, we acquired lipopolysaccharide (LPS) from Selleck.cn (China), consistent with a previous study^36^. The concentration of LPS utilized in this experiment was 2 μg/ml.

### Histological analysis of colitis

The colon tissues were immersed in a 4% paraformaldehyde solution for 36 h and then embedded in paraffin. Subsequently, 5 μm paraffin sections were prepared and subjected to hematoxylin and eosin staining. The extent of inflammation in the colonic tissues was evaluated using a light microscope at both 40 × and 100 × magnifications.

### RNA extraction and RT‑qPCR analysis

Samples were used for total RNA extraction using the RNA extraction kit. cDNA was synthesized with a reverse transcription kit (YEASEN,11141ES10). For quantitative PCR, the cDNA, Hieff UNICON ^®^Universal Blue Q-RCR SYBR Green Master Mix (YEASEN), and primers were combined. GAPDH was used as an internal reference gene, and the relative expression levels of the genes were calculated using the 2^−ΔΔCt^ method. The primers used in this study were as follows: IL-1β, forward: 5ʹ-ATGATGGCTTATTACAGTGGCAA-3ʹ, reverse: 5ʹ-GTCGGAGATTCGTAGCTGGA-3ʹ; IL-6, forward: 5ʹ-ACTCACCTCTTCAGAACGAATTG-3ʹ, reverse: 5ʹ-CCATCTTTGGAAGGTTCAGGTTG-3ʹ; TNF-α: forward: 5ʹ-CCTCTCTCTAATCAGCCCTCTG-3ʹ, reverse: 5ʹ-GAGGACCTGGGAGTAGATGAG-3ʹ; GAPDH, forward: 5ʹ-ACCCAGAAGACTGTGGATGG-3ʹ, reverse: 5ʹ-TCAGCTCAGGGATGACCTTG-3ʹ; GLS, forward: 5ʹ-AGGGTCTGTTACCTAGCTTGG-3ʹ, reverse: 5ʹ-ACGTTCGCAATCCTGTAGATTT-3ʹ.

### Western blot analysis

Inflammatory and normal colorectal tissue samples were added to RIPA lysis buffer with PMSF and homogenized on ice using a tissue grinder. The supernatant proteins were then extracted by centrifugation after 30 min of lysis and quantified using a BCA protein assay kit (Beyotime, Shanghai, China). Subsequently, 20 µg of proteins were separated using SDS–polyacrylamide gel electrophoresis and transferred to a polyvinylidene fluoride membrane. Following incubation with 5% skimmed milk for 1 h at room temperature, the membranes were exposed to primary antibodies (anti-GLS, anti-COX2, anti-IL-1β, anti-IL-10, anti-TNF-α, and anti-β-actin; proteintech, Wuhan, China) at 4 °C overnight (12–16 h). Later, the membranes were incubated with HRP-conjugated secondary antibodies and detected using an ECL chemiluminescence kit (Thermo Fisher). Protein quantification was then performed by normalizing to β-actin using ImageJ software.

### Statistical analysis

In this study, we performed all statistical analyses using R software version 4.3.2 and GraphPad Prism 8.0. We used the Wilcoxon rank-sum test to assess for significant differences between the two groups, with a p-value < 0.05 considered statistically significant.

### Ethics approval

All animal experiments were conducted in accordance with the ARRIVE guidelines. It was approved by the Ethics Committee of the First Affiliated Hospital of Nanchang University (Ethical number: (2022) CDYFYYLK (06-025)). All procedures were performed in accordance with relevant guidelines and regulations.

## Results

**Figure 1:**
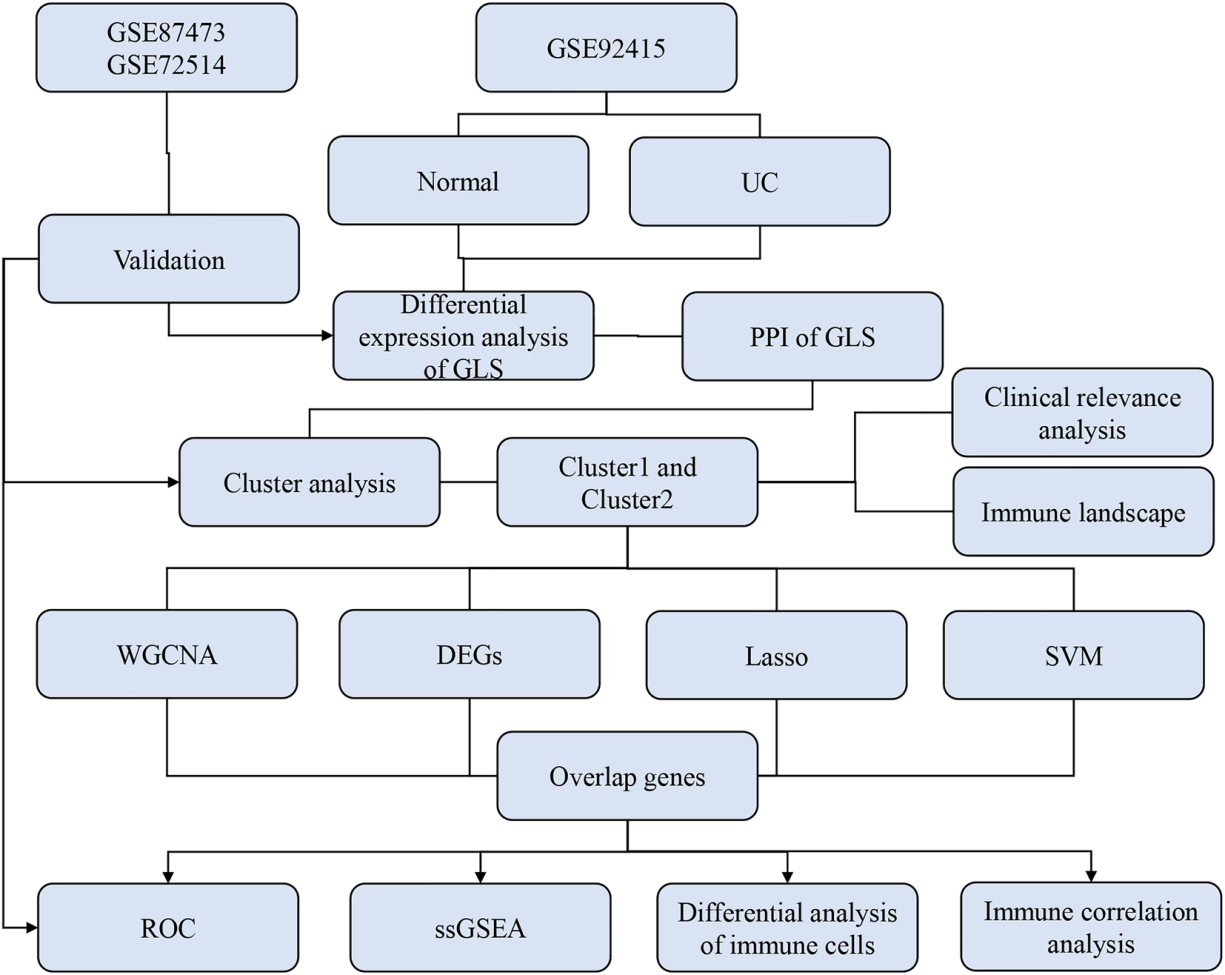
Flowchart of study design.

The flowchart of the study design is presented in Fig. 1.

### Identification of GLS expression and its related genes in UC

**Figure 2:**
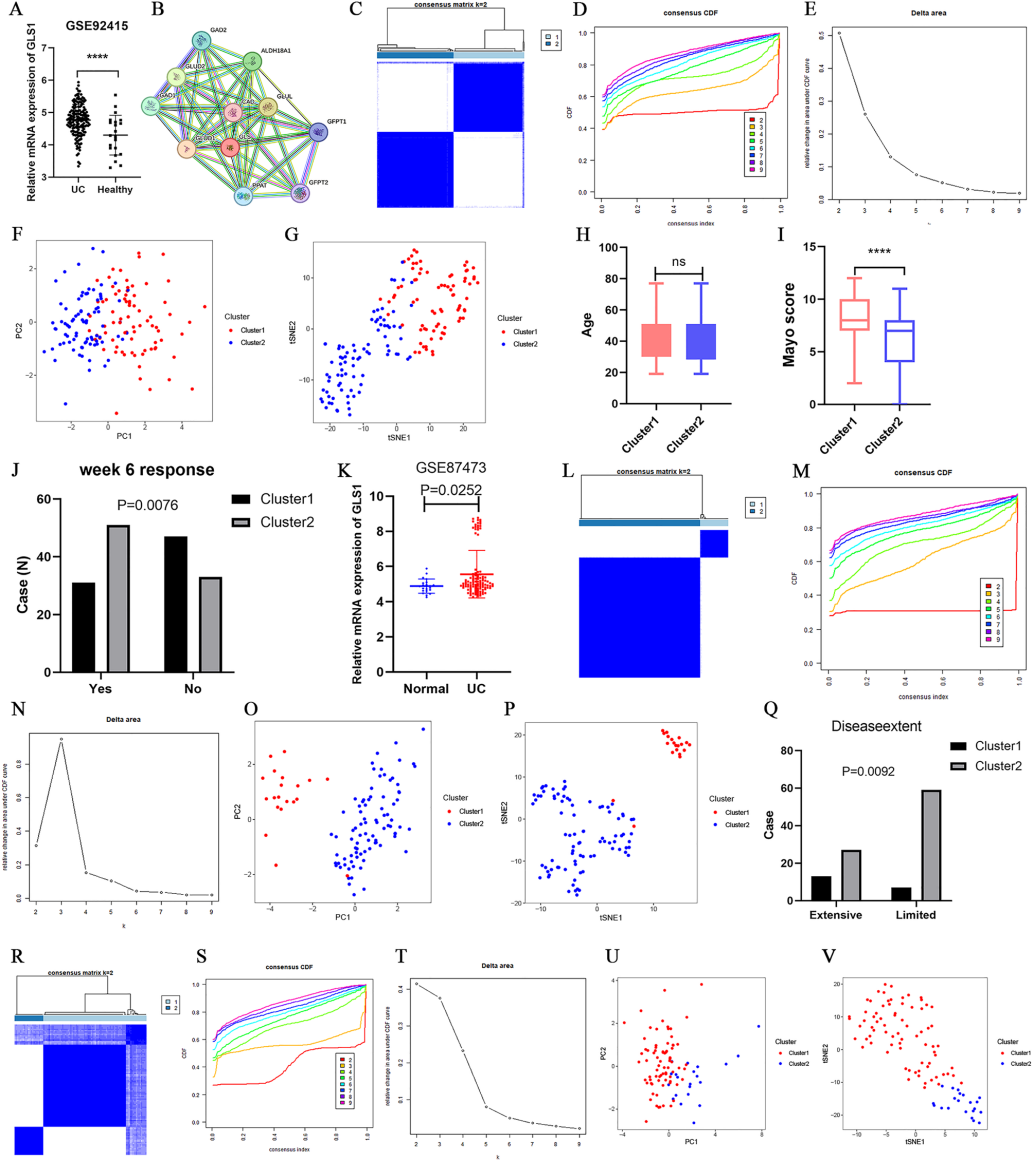
Consensus clustering analysis was performed for UC patients. The expression of GLS was examined in GSE92415 (**A**) and GSE87473 (**K**); (**B**) Schematic diagram of GLS protein interactions; Consensus clustering matrix for the two UC subtypes in GSE92415 (**C**), GSE87473 (**L**), and GSE72514 (**R**); The CDF curves from k = 2 to 10 in GSE92415 (**D**), GSE87473 (**M**), and GSE72514 (**S**). The relative change in area under cumulative distribution function (CDF) curve from k = 2–10 in GSE92415 (**E**), GSE87473 (**N**), and GSE72514 (**T**); PCA distribution plot for the UC subtype in GSE92415 (**F**), GSE87473 (**O**), and GSE72514 (**U**); t-SNE plot of Cluster1 and Cluster2 in GSE92415 (**G**), GSE87473 (**P**), and GSE72514 (**V**); (**H**) Relationship between subclusters and age in GSE92415; (**I**) Relationship between subclusters and mayo score in GSE92415; (**J**) Relationship between subclusters and placebo or golimumab 6-week response in GSE92415; (**Q**) Relationship between subclusters and disease extent in GSE87473.

We identified significantly higher expression of GLS1 in UC patients compared to the healthy population in the GSE92415 dataset (Fig. 2A). The proteins interacting with GLS1 were obtained through protein interaction analysis and are shown in Fig. 2B. Consensus clustering analysis based on GLS1 interaction-related genes was performed to classify patients with UC in the GEO training cohort into two subtypes (Fig. 2C–E). The PCA and t-SNE plot demonstrated complete distinction of 162 UC patients into the two subtypes (Fig. 2F–G). Analysis of the typing and clinical data revealed a higher Mayo score for Cluster 1 and no significant difference in age between Cluster 1 and Cluster 2 (Table 1, Fig. 2H,I). Moreover, the number of non-responding patients was higher in the Cluster 1 group after 6 weeks of placebo or golimumab treatment (Fig. 2J, P = 0.0076). The difference in GLS1 expression in normal and UC patients in the GSE87473 dataset was further validated (Fig. 2K). The typing of UC patients using GLS1-related genes resulted in an equal division into two types (Fig. 2L–N). The PCA and t-SNE plot revealed complete division of 106 UC patients into two subtypes (Fig. 2O,P). The UC patients in the Cluster 1 group presented a wider range of intestinal lesions, while those in the Cluster 2 group had a more limited range of intestinal lesions (Fig. 2Q). Additionally, upon validation in dataset GSE72514, the GLS1-associated genes successfully stratified UC patients into two distinct subtypes (Fig. 2R–T). PCA and t-SNE plots demonstrated clear segregation of 97 UC patients into two distinct groups (Fig. 2U,V).

**Table 1:** Patient demographics and baseline characteristics in different UC subtypes.

Characteristic	UC patients		p-value^b^
	Cluster1, N = 78^a^	Cluster2, N = 84^a^	
Age	42 ± 14	42 ± 14	0.851
Mayo score	8.00 ± 2.24	6.02 ± 2.82	< 0.001
Treatment			0.620
Golimumab	51 (65.4%)	58 (69.0%)	
Placebo	27 (34.6%)	26 (31.0%)	
Week 6 response			0.008
Yes	31 (39.7%)	51 (60.7%)	
No	47 (60.3%)	33 (39.3%)	

^a^Mean ± SD; n (%).

^b^Welch Two sample t-test; Pearson's Chi-squared test.

### Identification of differential expression genes between C1 and C2 subtypes

**Figure 3:**
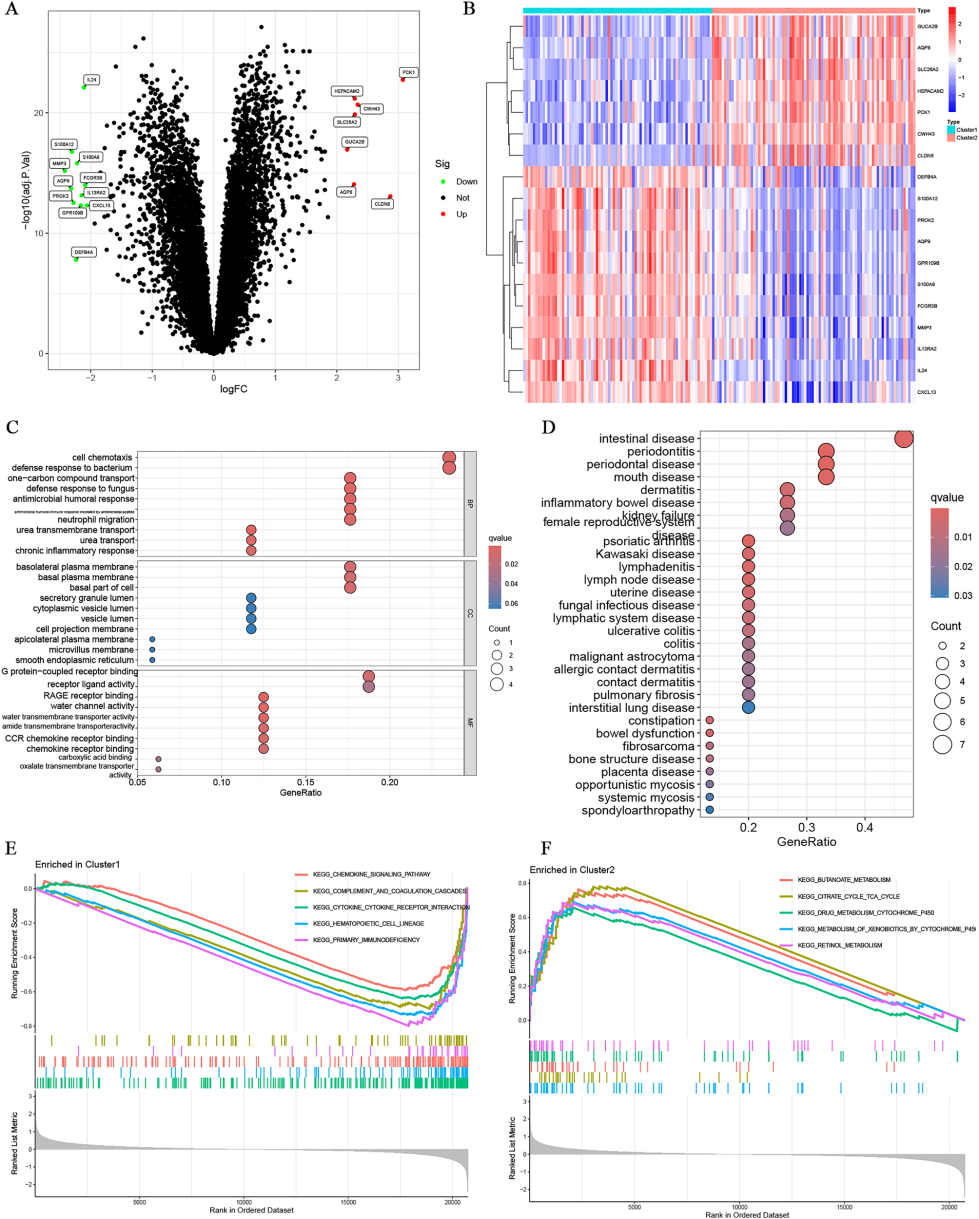
DEGs signature in UC patients. (**A**) Volcano plot showing differentially expressed genes between Cluster1 and Cluster2; (**B**) Heatmap of differentially expressed genes; (**C**) Gene Ontology (GO) analysis of DEGs; (**D**) Disease Ontology (DO) analysis of DEGs; GSEA between Cluster1 (**E**) and Cluster2 (**F**).

A total of 18 differentially expressed genes (DEGs) were identified between the C1 and C2 subtypes, meeting the criteria |log2(fold change) |≥ 2 and FDR < 0.05, including 7 upregulated genes and 11 downregulated genes. A volcano plot and heatmap of the DEGs and their expressions were generated and presented (Fig. 3A,B). The DEGs were then extracted for enrichment analyses using the DAVID database. Figure 3C depicts the thirty most highly enriched terms for gene ontology (GO) analysis, including biological pathways (BP), cytological components (CC), and molecular functions (MF). The top five enriched BP terms were cell chemotaxis, defense response to bacterium, one-carbon compound transport, defense response to fungus, and antimicrobial humoral response. The three most significant enriched CC terms included basolateral plasma membrane, basal plasma membrane, and basal part of the cell. The eight most enriched MF terms were G protein-coupled receptor binding, receptor ligand activity, RAGE receptor binding, water channel activity, water transmembrane transporter activity, amide transmembrane transporter activity, CCR chemokine receptor binding, and chemokine receptor binding. Figure 3D depicts the thirty most highly enriched terms for Disease Ontology (DO) analysis. The most significant enriched DO terms included intestinal disease, along with diseases such as ulcerative colitis and colitis. To assess the differences in signaling pathways between subtypes of UC patients, a gene set enrichment analysis (GSEA) was performed. The results showed that the chemokine signaling pathway, complement and coagulation cascades, cytokine-cytokine receptor interaction, hematopoietic cell lines, and primary immunodeficiencies were activated in all cluster 1 subtypes (Fig. 3E). Meanwhile, butanoate metabolism, citrate cycle TCA cycle, drug metabolism cytochrome p450, metabolism of xenobiotics by cytochrome p450, and retinol metabolism were activated in all cluster 2 subtypes (Fig. 3F).

### Characteristic biomarker identification in the UC training cohort

**Figure 4:**
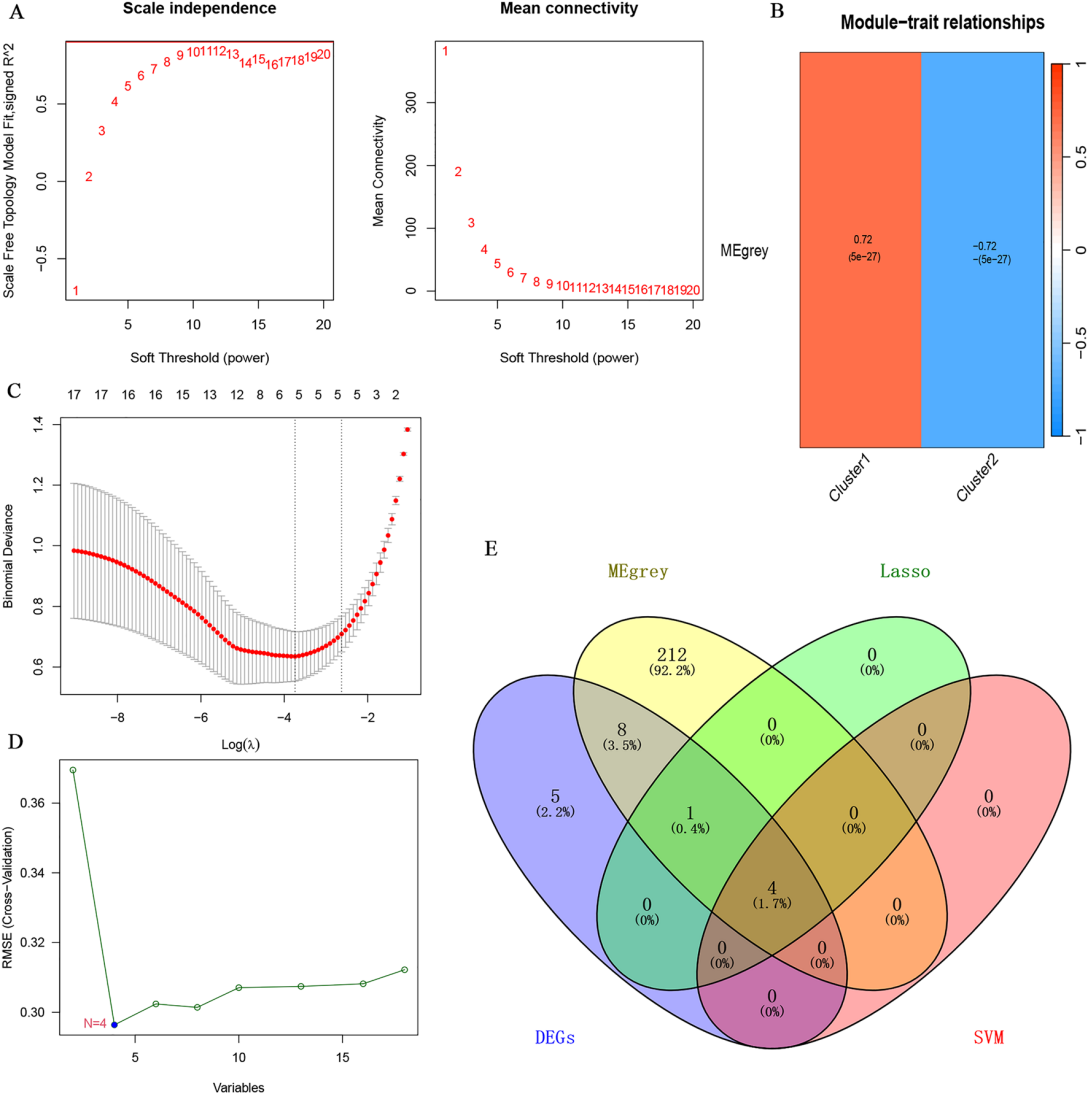
Identification of characteristic biomarkers in the UC training cohort. (**A**) Analysis of soft thresholding power in WGCNA; (**B**) Characteristic association of modules; (**C**) Screening of characteristic biomarkers for UC using the LASSO regression algorithm; (**D**) Identification of characteristic biomarkers for UC using the SVM-RFE machine-learning algorithm; (**E**) Intersection of genes among DEGs, WGCNA, LASSO regression algorithm, and SVM-RFE algorithm.

In the GEO training cohort, we identified the most relevant module in the subtype as ME_grey through WGCNA analysis (Fig. 4A,B). A total of 225 feature genes were included in the analysis (Fig. 4E). Additionally, the LASSO regression algorithm identified 5 feature genes (Fig. 4C,E), while the SVM-RFE algorithm identified 4 feature genes (Fig. 4D,E). The Venn plot illustrated the overlapping feature genes in UC patients among the 3 algorithms (Fig. 4E; CWH43, HEPACAM2, IL24, PCK1).

### Characteristic biomarker validation in the UC training cohort and validation cohort

**Figure 5:**
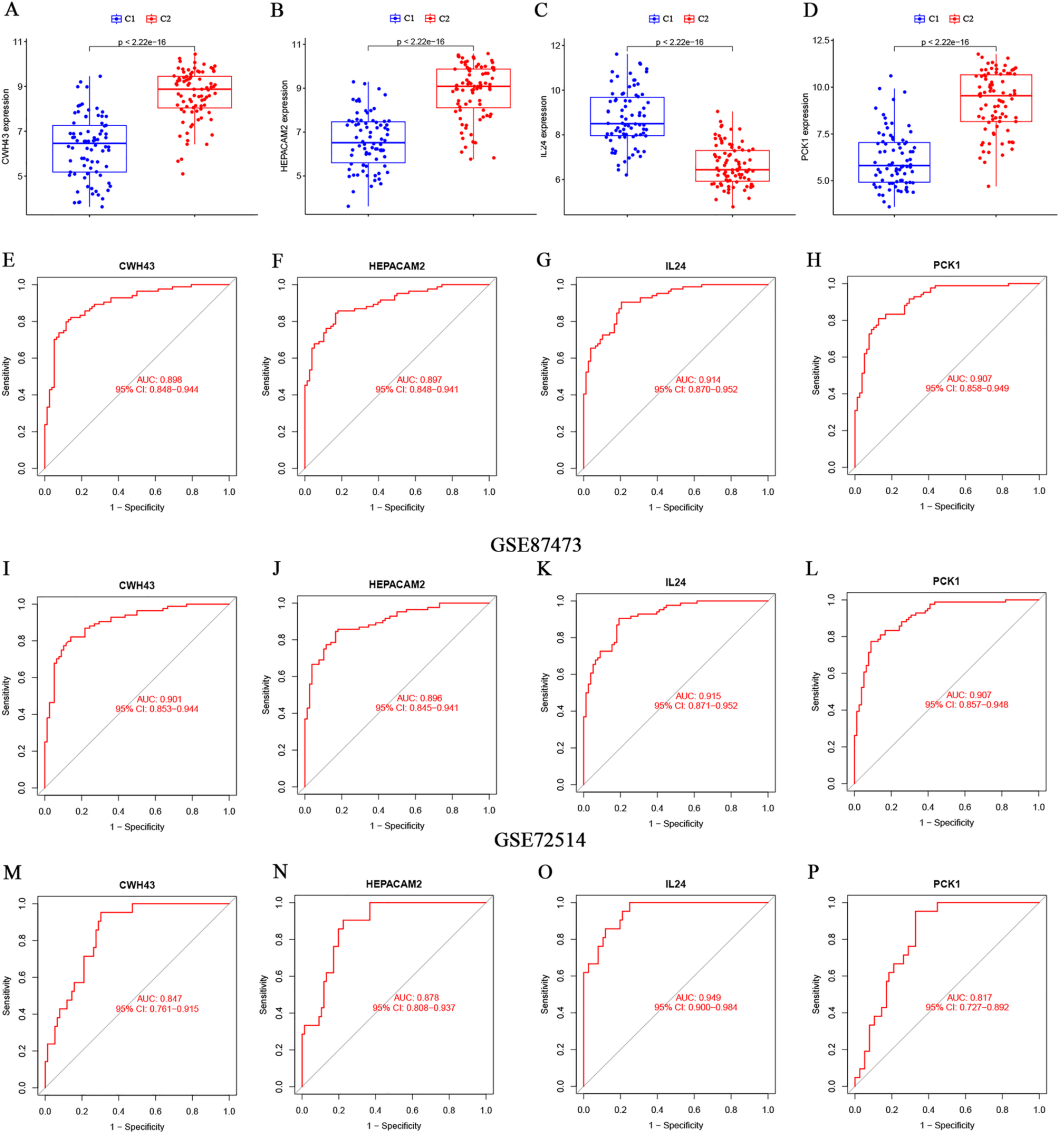
(**A**–**D**) The expression levels of the characteristic genes in the training cohort. ROC curves were used to evaluate the diagnostic accuracy of the characteristic genes in both the training cohort (**E**–**H**) and the validation cohort (GSE87473 **I**–**L**, GSE72514 **M**–**P**).

Among these four signature genes, the expression levels of CWH43, HEPACAM2, and PCK1 were significantly higher in patients with UC subtype C2, and the expression level of IL24 was significantly increased in patients with UC subtype C1 (Fig. 5A–D, p < 0.0001). ROC curves were plotted to evaluate the diagnostic accuracy of the CWH43, HEPACAM2, IL24, and PCK1 signature genes in discriminating the two subtypes (Fig. 5E–P). The results in Fig. 5E–H showed that CWH43, HEPACAM2, IL24, and PCK1 could be effective biomarkers for diagnosing UC subtypes (CWH43: AUC = 0.898, 95% CI 0.848–0.944; HEPACAM2: AUC = 0.897, 95% CI 0.848–0.941; IL24: AUC = 0.914, 95% CI 0.870–0.952; PCK1: AUC = 0.907, 95% CI 0.858–0.949). The diagnostic efficacy of CWH43, HEPACAM2, IL24, and PCK1 was further validated in the validation cohort (Fig. 5I–L, CWH43: AUC = 0.901, 95% CI 0.853–0.944; HEPACAM2: AUC = 0.896, 95% CI 0.845–0.941; IL24: AUC = 0.915, 95% CI 0.871–0.952; PCK1: AUC = 0.907, 95% CI 0.857–0.948). Additionally, the diagnostic potential of CWH43, HEPACAM2, IL24, and PCK1 was validated in dataset GSE72514 (Fig. 5M–P, CWH43: AUC = 0.847, 95% CI 0.761–0.915; HEPACAM2: AUC = 0.878, 95% CI 0.808–0.937; IL24: AUC = 0.949, 95% CI 0.900–0.984; PCK1: AUC = 0.817, 95% CI 0.727–0.892).

### Immune-infiltrating landscape of UC in training cohort

**Figure 6:**
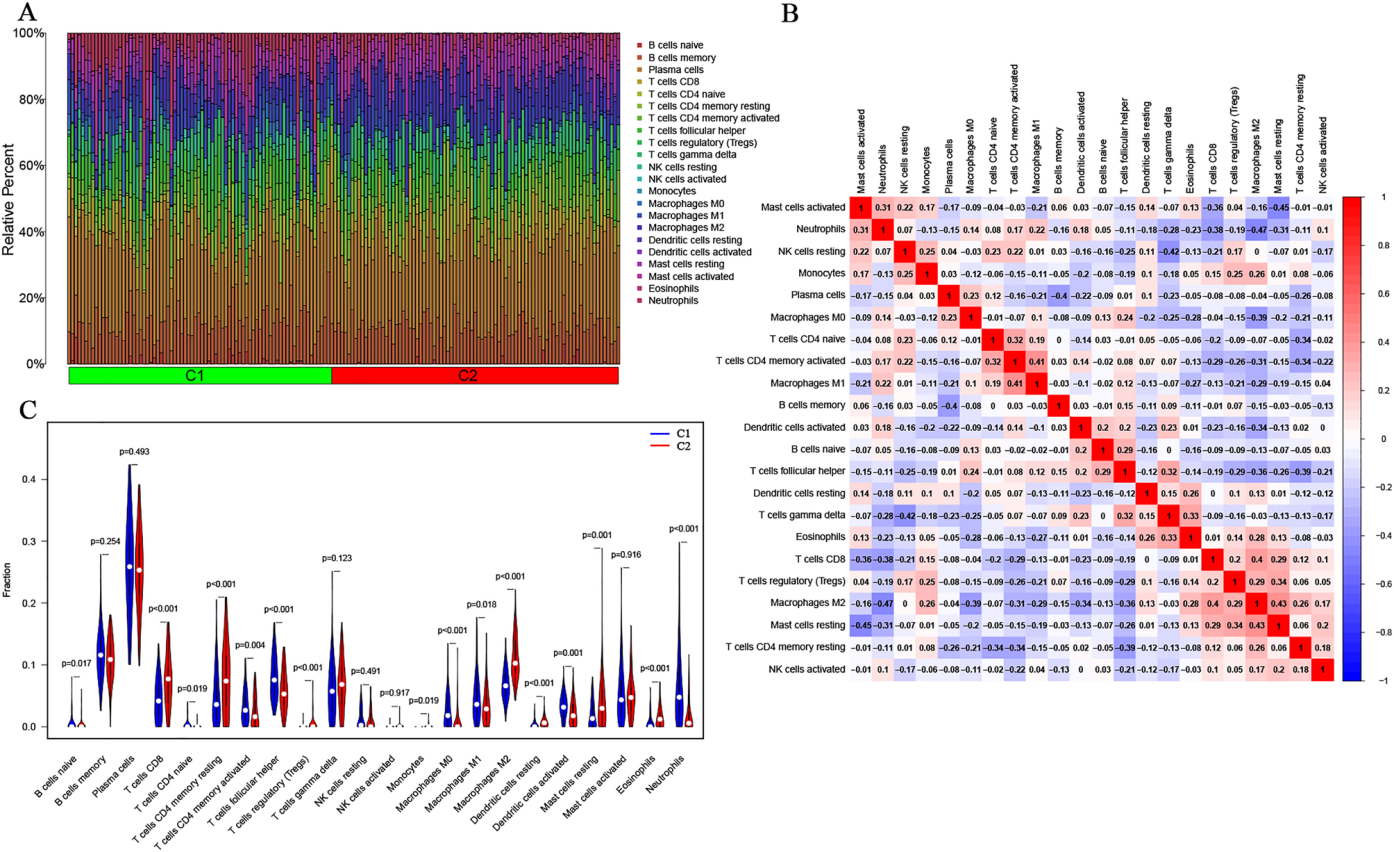
Proportion of immune cells in UC patients and their correlation analysis. (**A**) Percentage abundances of different immune cells in each sample of UC were determined by CIBERSORT. (**B**) Correlation matrix of different infiltrating immune cells in UC. The darker the blue, the stronger the negative correlation, and the darker the red, the stronger the positive correlation. (**C**) Comparison of immune cell infiltration in the intestinal mucosa between C1 subtype UC patients and C2 subtype UC patients. Blue represents the C1 group, and red represents the C2 group.

UC is a chronic inflammatory intestinal barrier disease, and mucosal immune dysregulation may be associated with the pathogenesis of UC^37^. Therefore, to elucidate the differences in immune cells between C1 and C2 UC subtypes, we utilized the CIBERSORT algorithm to measure the infiltration of 22 types of immune cells and compared them between the C1 and C2 subtypes in the training set. The composition of the immune cell population in each sample of the C1 subtype and C2 subtype UC was visualized in histograms (Fig. 6A). A weak correlation was observed between the components of different immune cell infiltrations (Fig. 6B). The M2 type of macrophage was found to be slightly more closely related to other immune cells. It was weakly negatively correlated with Neutrophils (r = − 0.47) and M0 macrophages (r = − 0.39), and weakly positively correlated with T cells CD8 (r = 0.4) and Mast cells resting (r = 0.43). The difference in abundance of immune cell infiltration in the intestinal mucosa between the C1 and C2 subtypes in UC is depicted in Fig. 6C. Immune cells with higher levels of infiltration in the intestinal mucosa of patients with C1 UC compared to the C2 group included B cells naïve (p = 0.017), T cells CD4 naïve (p = 0.019), T cells CD4 memory activated (p = 0.004), T cells follicular helper (p < 0.001), M0 macrophages (p < 0.001), M1 macrophages (p = 0.018), activated dendritic cells (p = 0.001), and neutrophils (p < 0.001). However, the group of patients with C2 UC had higher levels of T cells CD8 (p < 0.001), T cells CD4 memory resting (p < 0.001), T cells regulatory (p < 0.001), Monocytes (p = 0.019), M1 macrophages (p < 0.001), Dendritic cells resting (p < 0.001), Mast cells resting (p = 0.001), and Eosinophils (p < 0.001).

### UC characteristic biomarkers are associated with the tumor immune microenvironment

**Figure 7:**
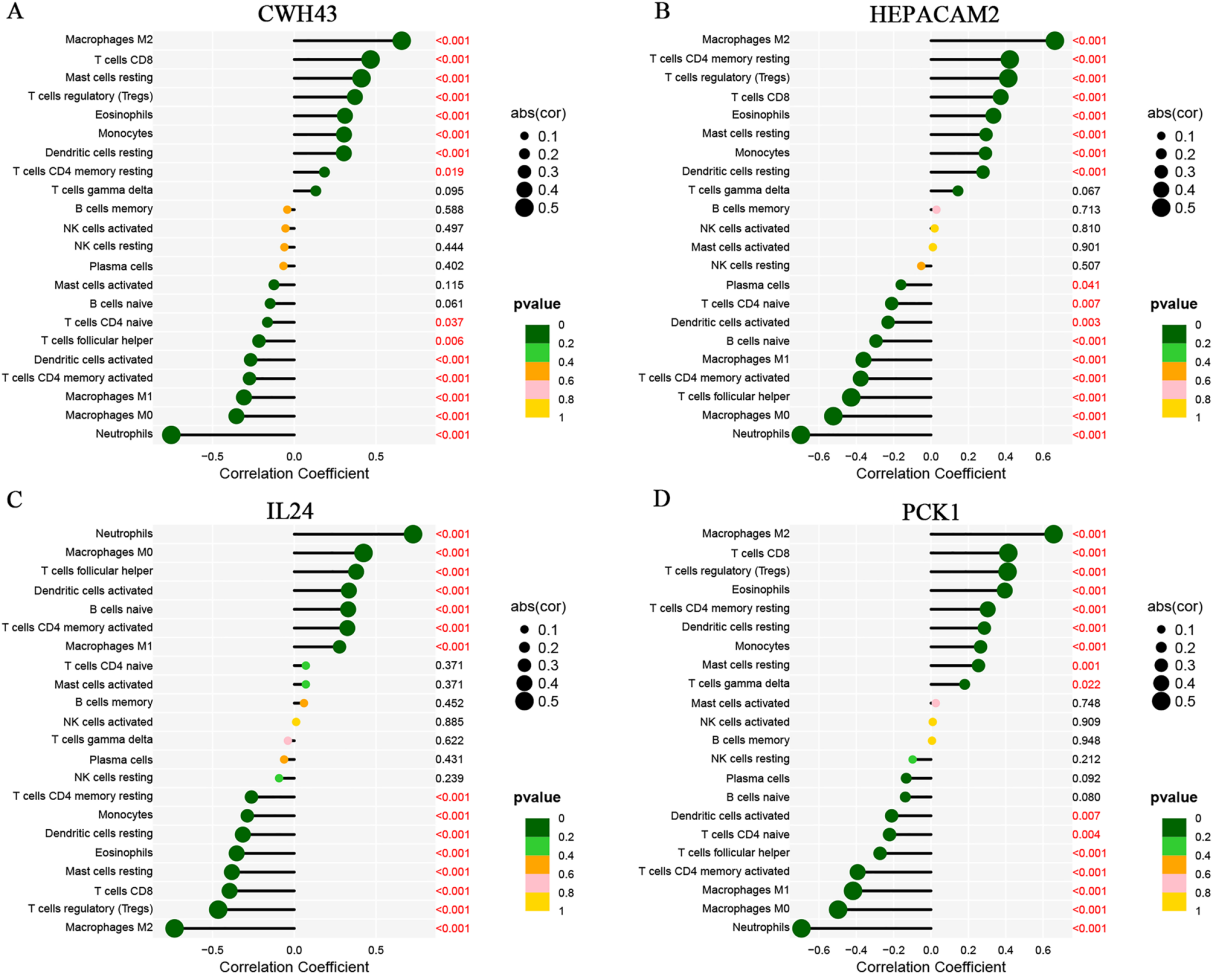
Relationship between the expression levels of characteristic biomarkers and infiltrating immune cells. (**A**) Correlation between the expression level of CWH43 and infiltrating immune cells. (**B**) Correlation between the expression level of HEPACAM2 and infiltrating immune cells. (**C**) Correlation between the expression level of IL24 and infiltrating immune cells. (**D**) Correlation between the expression level of PCK1 and infiltrating immune cells.

We investigated the correlation between immune cell infiltration in patients with ulcerative colitis (UC) and the expression levels of CWH43, HEPACAM2, IL24, and PCK1. The expression level of CWH43 was positively correlated with the infiltration levels of macrophage M2, CD8 T cells, resting mast cells, regulatory T cells, eosinophils, monocytes, resting dendritic cells, and memory resting CD4 T cells. Conversely, it demonstrated a negative correlation with naive CD4 T cells, follicular helper T cells, activated dendritic cells, activated memory CD4 T cells, macrophages M0, macrophages M1, and neutrophils (Fig. 7A, p < 0.05). HEPACAM2 expression displayed a positive correlation with the infiltration levels of macrophages M2, memory resting CD4 T cells, regulatory T cells, CD8 T cells, eosinophils, resting mast cells, monocytes, and resting dendritic cells. Conversely, it showed a negative correlation with plasma cells, naive CD4 T cells, activated dendritic cells, naive B cells, macrophages M1, activated memory CD4 T cells, follicular helper T cells, macrophages M0, and neutrophils (Fig. 7B, p < 0.05). The expression level of IL24 was positively correlated with neutrophils, macrophages M0, follicular helper T cells, activated dendritic cells, naive B cells, activated memory CD4 T cells, and macrophages M1. Conversely, it exhibited a negative correlation with memory resting CD4 T cells, monocytes, resting dendritic cells, eosinophils, resting mast cells, CD8 T cells, regulatory T cells, and macrophage M2 (Fig. 7C, p < 0.05). PCK1 expression level demonstrated a positive association with macrophages M2, CD8 T cells, regulatory T cells, eosinophils, memory resting CD4 T cells, resting dendritic cells, monocytes, resting mast cells, and gamma delta T cells. Conversely, it showed a negative correlation with activated dendritic cells, naive CD4 T cells, follicular helper T cells, activated memory CD4 T cells, macrophages M1, macrophages M0, and neutrophils (Fig. 7D, p < 0.05).

### Validation of GLS expression in a cellular model of LPS-induced enteritis and an acute mouse model of colitis

**Figure 8:**
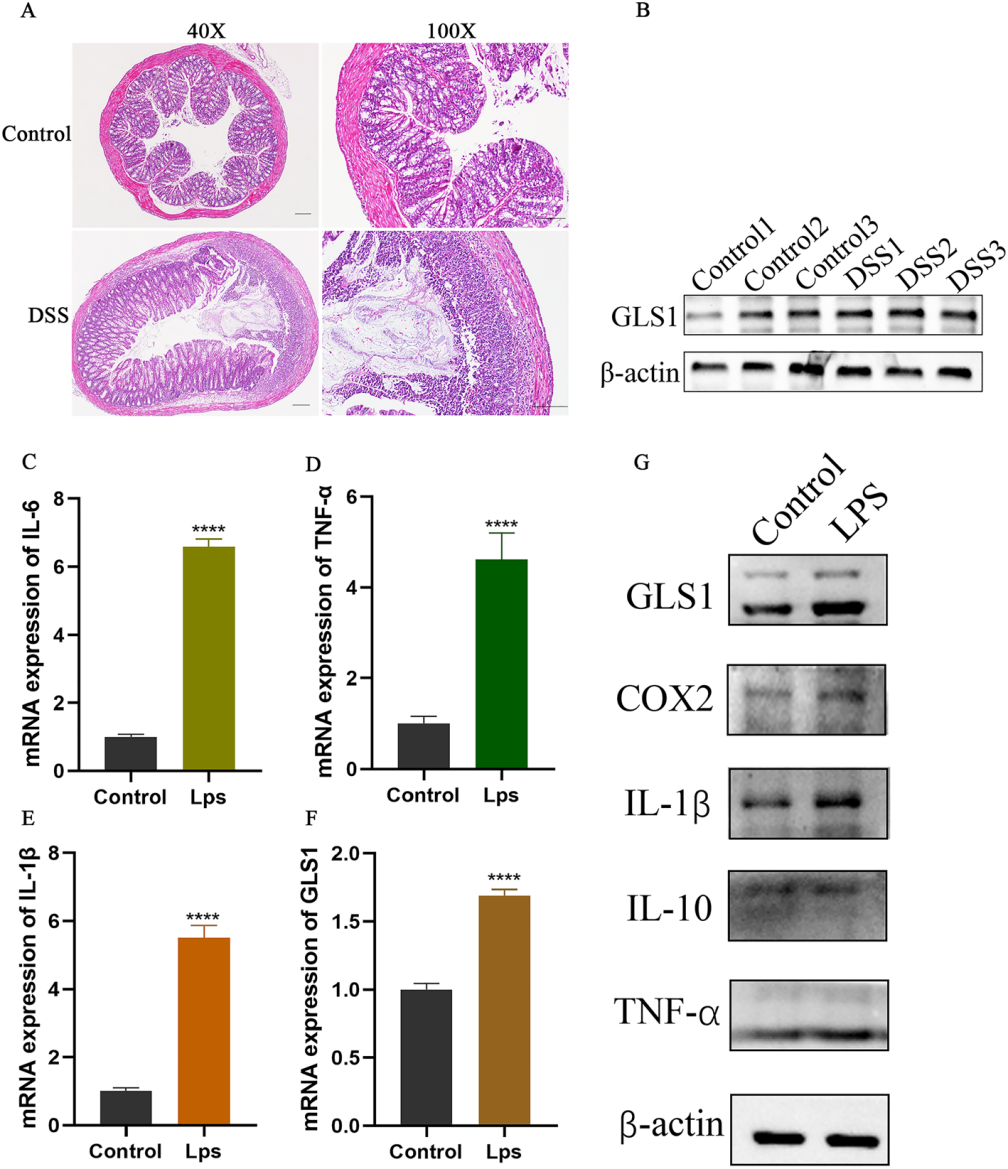
Cell and animal experiments validate GLS1 expression. (**A**) Hematoxylin and eosin staining of control and acute DSS mouse model intestines; (**B**) GLS protein expression in the intestines of control and ulcerative colitis mice; mRNA expression of inflammatory factors IL-6 (**C**), TNF-α (**D**), IL-1β (**E**), and GLS1 (**F**) in control and cellular inflammation model; (**G**) Protein expression of inflammatory factors in control and cellular inflammation model.

To validate the expression of GLS in UC, we conducted assessments on mouse enteritis tissues. We performed HE staining to confirm the successful modeling of UC in mice, as shown in Fig. 8A. Subsequently, we observed elevated expression of GLS in mouse intestinal inflammatory tissues, as depicted in Fig. 8B. Further validation was carried out in a cellular inflammation model. Figure 8C–E,G illustrate elevated expression of inflammatory factors in the NCM460 inflammatory cell model, confirming the successful establishment of the model. We then reassessed GLS expression, revealing elevated mRNA and protein levels in the NCM460 inflammatory cell model, as seen in Fig. 8F,G.

## Discussion

Ulcerative colitis is a chronic inflammatory bowel disease with an unclear etiology^38^. Treatment effectiveness and patient prognosis for UC vary from person to person according to guidelines. Some patients can maintain remission for an extended period, while others may experience periodic remissions and relapses^39^. It would be beneficial to classify UC based on specific molecular characteristics in order to predict disease state and prognosis for better patient management. Our study aimed to classify UC patients based on GLS-related genes, identify differences in disease severity and treatment efficacy among different UC subtypes, and identify characteristic biomarkers for clinical guidance in different typologies.

In this study, we acquired the GSE92415 dataset from the GEO database as the training set, comprising transcriptome sequencing data from 162 UC samples and 21 normal colon tissue samples. Additionally, we obtained the GSE87473 dataset as a validation set, encompassing transcriptome sequencing data from 106 UC samples and 21 normal colon tissue samples. GSE72514 dataset, comprises transcriptomic data collected from 97 UC patients’ colonic tissue samples, as another validation group. Our analysis revealed a significant up-regulation of GLS in UC samples across both datasets. We identified GLS interaction-related genes through the String database and conducted consistent clustering analysis on UC samples based on these genes, resulting in cluster1 and cluster2. Differential analyses between the two subtypes identified a total of 7 up-regulated and 11 down-regulated DEGs. Enrichment analyses indicated that these DEGs were primarily associated with cell chemotaxis, defense responses against bacteria, and were implicated in intestinal disorders and UC. Furthermore, GSEA analysis of C1 and C2 subtypes revealed distinct associations with crucial biological processes. C1 was linked to pathways such as chemokine signaling and cytokine-cytokine receptor interactions, while C2 showed associations with butyrate metabolism and the citric acid cycle TCA cycle. Chemokines play a pivotal role in various biological responses, including cell polarization, motility, and immune and inflammatory responses^40^. It has been found that chemokine receptors can be used as therapeutic targets for tumors^41^. In a study by Chang et al., chemokine ligand 14 (CXCL14) was found to promote metastasis in lung cancer as a prognostic biomarker through an ACKR2-dependent signaling pathway^42^. Notably, dysregulated expression of molecules involved in pro- and anti-inflammatory processes has been associated with UC pathogenesis. For instance, the chemokine CXCL8, a crucial pro-inflammatory factor in UC^43^, influences the disease through multiple signaling pathways, including PI3K/Akt, MAPKs, and NF-κB pathways^44–46^. This dysregulation may contribute to the elevated disease activity and poorer prognosis observed in patients with the C1 subtype of UC. On the other hand, butyrate, a colonic metabolite of carbohydrates, emerged as a significant factor associated with the C2 subtype. Butyrate is considered a major energy source for the colonic mucosa and has been found to inhibit the growth of colorectal cancer^47^. Studies also suggest that butyrate can alleviate enteritis induced by DSS or Citrobacter in mice^48^, providing potential explanations for the better prognosis and treatment outcomes observed in patients with C2 subtype UC. Therefore, in the future treatment of UC, for UC patients with type C1, whether we can consider the treatment with butyrate and chemokine inhibitors to reduce the patient's disease activity state remains to be further clinically verified.

We utilized WGCNA, SVM-RFE, and LASSO regression machine learning algorithms to identify signature genes for UC, including CWH43, HEPACAM2, IL24, and PCK1. The Cell Wall Biogenesis 43 C-Terminal Homolog (CWH43) promotes colorectal tumor cell growth by enhancing TTK-mediated cell cycle activity^49^. Interestingly, patients with C1 subtype UC displayed higher Mayo scores and were less responsive to the 6-week treatment (Fig. 2I,J). CWH43 expression was lower in C1 subtype UC (Fig. 5A), suggesting a potential link between CWH43 and disease severity, warranting further investigation. HEPACAM2, a member of the HEPACAM family, encodes a protein related to the immunoglobulin superfamily, functioning in mitosis. HEPACAM has been reported to be down-regulated in breast cancer and induces senescence-like growth arrest by increasing the expression levels of senescence-associated proteins p21, p27 and p53^50^. HEPACAM has been reported to be down-regulated in breast cancer and induces senescence-like growth arrest by increasing the expression levels of senescence-associated proteins p21, p27 and p53^50^. It has also been implicated as an oncogene in various tumors, including bladder cancer^51^, prostate cancer^52,53^, renal clear cell carcinoma^54^. Interleukin 24 (IL24), an immunomodulatory cytokine, exhibits cancer-specific inhibitory effects such as angiogenesis inhibition, sensitivity to chemotherapy, and cancer-specific apoptosis^55^. IL24 belongs to the IL-20 subfamily, participating in host defense against bacteria and fungi, tissue remodeling, and wound healing. Elevated IL24 expression has been reported in active UC^56^. In GO functional enrichment analysis, cell chemotaxis, defense response against bacteria, defense response against fungi, and antimicrobial humoral response differed across UC subtypes, corresponding to the results of previous studies (Fig. 3C). Phosphoenolpyruvate carboxykinase1 (PCK1), the first rate-limiting enzyme in gluconeogenesis, plays a crucial role in glucose homeostasis and adipogenesis^57^. PCK1 not only regulates glucose homeostasis but also regulates adipogenesis through activation of sterol regulatory element-binding proteins^58^. Ye et al. found that deficiency of PCK1 leads to metabolic-associated fatty liver disease^59^, and promotes CHK2 O-GlcNAcylation and hepatocellular carcinoma growth in glucose deficiency^60^. In our study, we found that PCK1 expression was decreased in patients with C1 subtype UC, and further studies are needed to determine whether PCK1 is a potential cause of its poorer prognosis. Guidelines recommend fecal calprotectin, fecal lactoferrin, and CRP for activity monitoring and therapeutic decision-making in UC^61^. There are no other validated molecular biomarkers for UC typing and activity monitoring. Interestingly, our study found that the expression of CWH43, HEPACAM2, IL24, and PCK1 could better identify the 2 subtypes of UC (Fig. 5E–L) and established a link with UC for the first time.

We investigated the relationship between UC signature genes and immune cell infiltration using the CIBERSORT algorithm. Many studies have shown that the immune microenvironment is associated with the pathogenesis of UC^62^. Mitsialis et al. found significant differences in specific immune cell populations in mucosal and blood samples from UC patients and control patients^63^. Significant changes in CD 8 T cells in UC have been observed by single-cell analysis^64^. In our study, the proportion of seven immune cells was significantly higher in patients with C2 subtype UC than in patients with C1 subtype UC, including M2 macrophages, CD8 T cells, and regulatory T cells. It further suggests the role of CD 8 T cells in different UC subtypes. In addition, there was a positive regulatory relationship between the UC signature genes CWH43, HEPACAM2, and PCK1 and M2 macrophages, CD8 T cells, and regulatory T cells. And there was a negative regulatory relationship between IL24 and these immune cells. It is suggested that CWH43, HEPACAM2, IL24 and PCK1 may be involved in the development of different subtypes of UC through immune regulation. Of course, these speculations require further studies to verify the role of immune response through gene integration regulation in different UC subtypes.

We explored the relationship between UC signature genes and immune cell infiltration using the CIBERSORT algorithm. Numerous studies have demonstrated that the immune microenvironment is linked to the pathogenesis of UC^55^. Mitsialis et al. identified significant differences in specific immune cell populations in mucosal and blood samples from UC patients and control patients^56^. Single-cell analysis has also revealed significant changes in CD8 T cells in UC^57^. In our study, we found that the proportion of seven immune cells was significantly higher in patients with C2 subtype UC compared to patients with C1 subtype UC, including M2 macrophages, CD8 T cells, and regulatory T cells. This suggests a potential role of CD8 T cells in different UC subtypes. Additionally, we identified a positive regulatory relationship between the UC signature genes CWH43, HEPACAM2, and PCK1 and M2 macrophages, CD8 T cells, and regulatory T cells, while there was a negative regulatory relationship between IL24 and these immune cells. This implies that CWH43, HEPACAM2, IL24, and PCK1 may be involved in the development of different subtypes of UC through immune regulation. However, these speculations require further studies to verify the role of immune response through gene integration regulation in different UC subtypes.

Finally, we examined the expression of the GLS gene using RT-qPCR and WB. The results of the WB showed a significant increase in the expression of GLS in the UC mouse model of enteritis. Similarly, GLS levels were elevated in the enteritis cell model. However, our study has some limitations. Firstly, we were unable to assess the prognostic value of different UC signature genes due to the lack of corresponding clinical information and outcome data in the GEO dataset. Secondly, there was a lack of external experiments to verify the expression and role of UC signature genes in different subtypes and specific mechanisms. To address these limitations, a prospective cohort study could be conducted to assess the prognostic value of the signature genes in predicting disease progression, response to therapy, and patient prognosis through the long-term collection of clinical data and gene expression profiles of UC patients. In addition, in vitro and in vivo experiments using cell lines, organoids, and animal models or patient samples are used to validate the expression and functional roles of UC signature genes.

## Conclusions

By integrating multiple bioinformatics tools and using GLS-related genes to characterize different subtypes, related pathways, and immune cell infiltration in UC tissues, we screened CWH43, HEPACAM2, IL24, and PCK1 as possible candidate diagnostic biomarkers for UC typing. These results will help to understand the pathogenesis of UC and provide new therapeutic tools.

### Supplementary Information

Supplementary Information.

## Data Availability

The data that endorse the results of this research are available upon reasonable request from the corresponding authors (CYZ and YXC).
