# Supplementary figures and images for: Identification of subclusters and prognostic genes based on GLS-associated molecular signature in ulcerative colitis
Source: Sci Rep. 2024 Jun 7;14:13102. doi: 10.1038/s41598-024-63891-2 (PMC11161595; doi:10.1038/s41598-024-63891-2)

Figure 8B

Marker

Con1  
Con2  
Con3  
DSS1  
DSS2  
DSS3

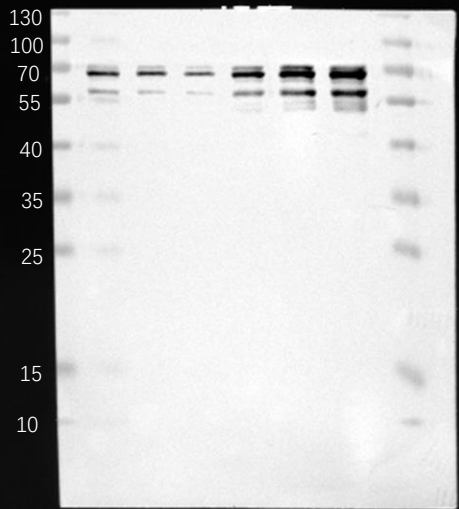

GLS1

Marker

Con1  
Con2  
Con3  
DSS1  
DSS2  
DSS3

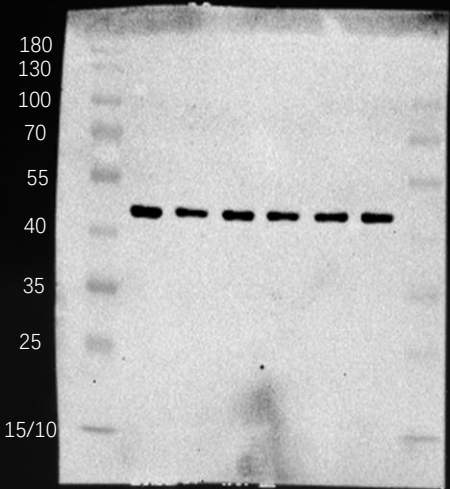

$\beta$ -actin

Figure 8G

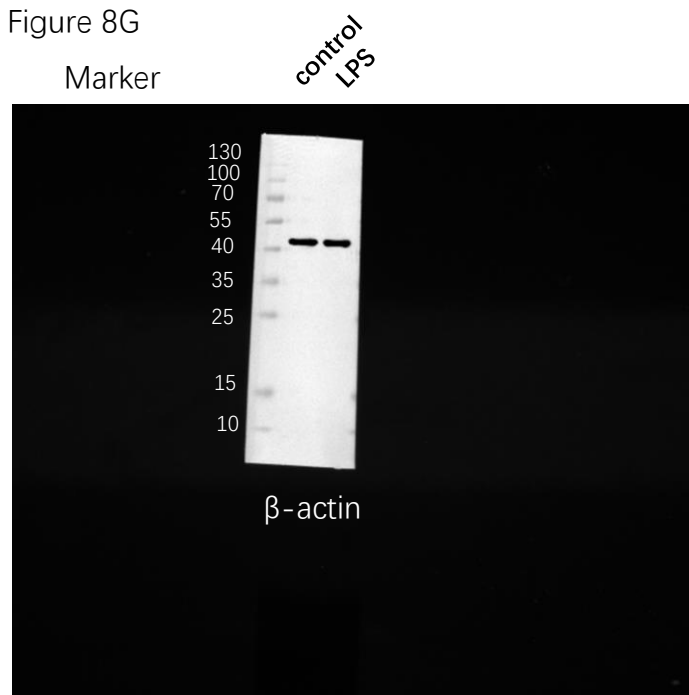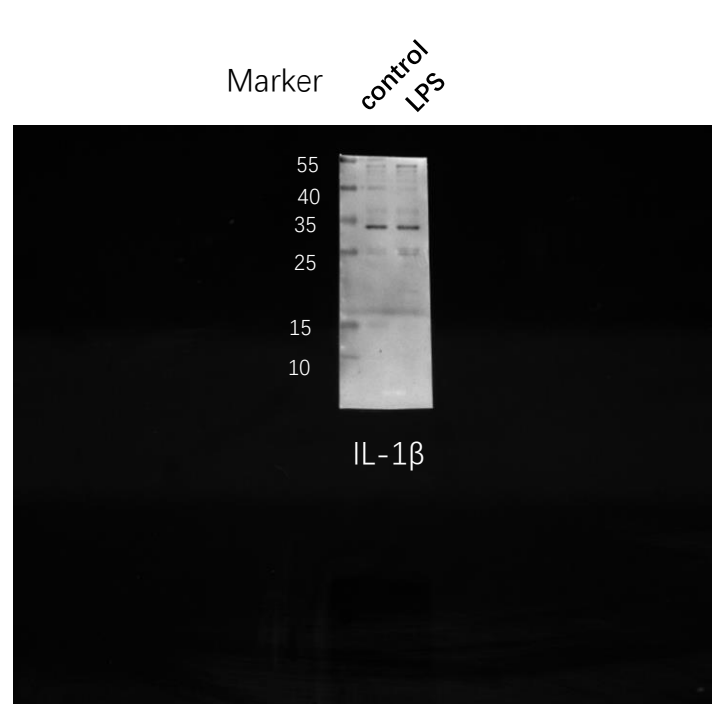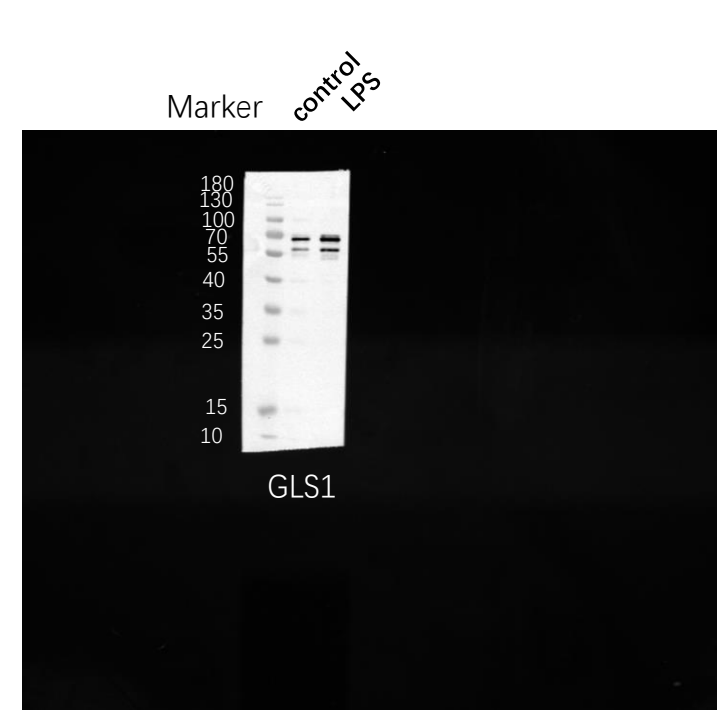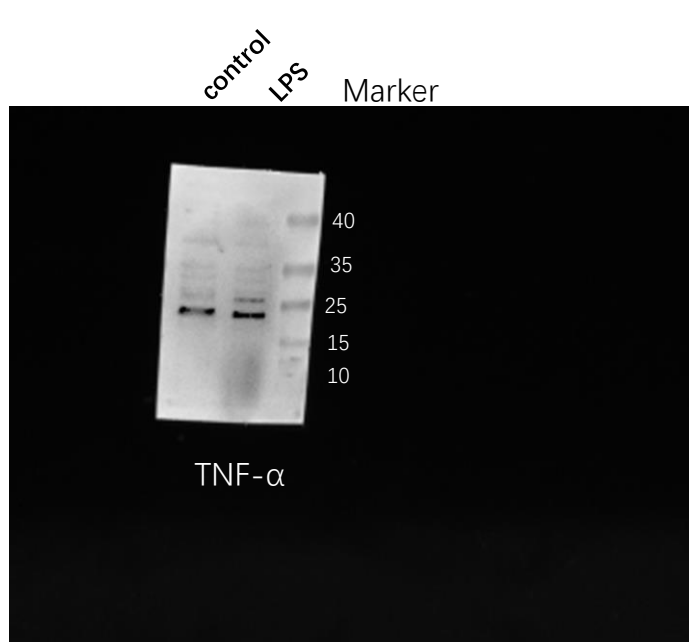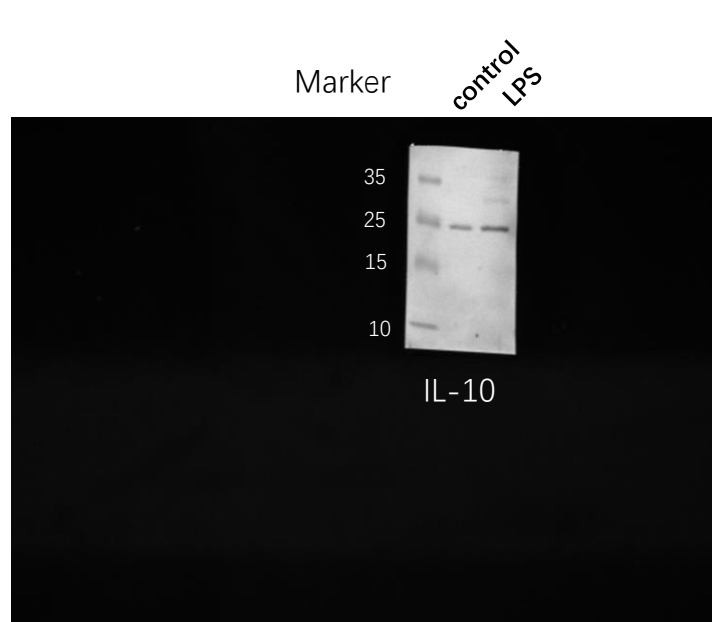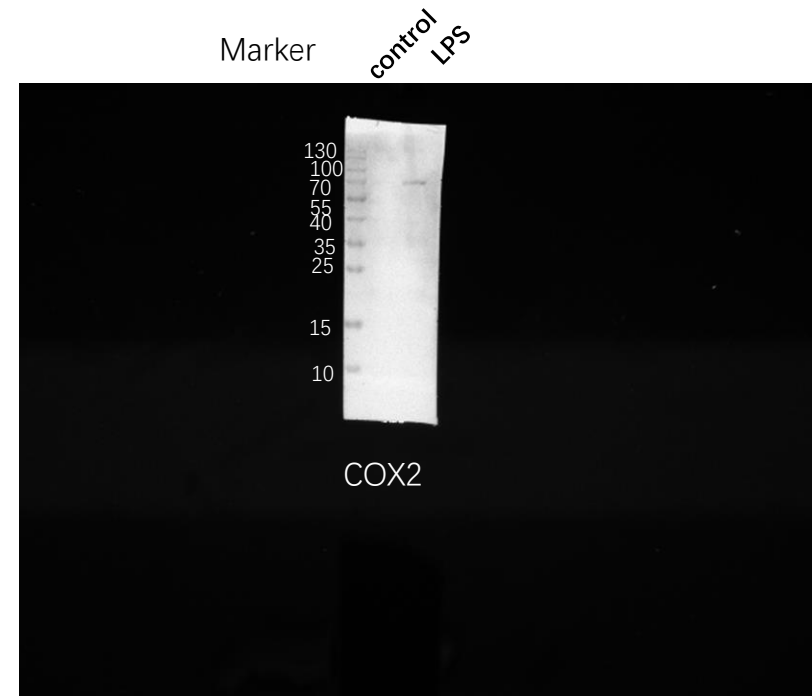

Supplement: Supplementary file 1 — Supplementary Information. [file 41598_2024_63891_MOESM1_ESM.pdf]
